# Hyperlactataemia is a marker of reduced exercise capacity in heart failure with preserved ejection fraction

**DOI:** 10.1002/ehf2.14794

**Published:** 2024-05-02

**Authors:** Emilia Nan Tie, Emil Wolsk, Shane Nanayakkara, Donna Vizi, Justin Mariani, Jacob Eifer Moller, Christian Hassager, Finn Gustafsson, David M. Kaye

**Affiliations:** ^1^ Department of Cardiology Alfred Hospital Melbourne VIC 3004 Australia; ^2^ Department of Cardiology Herlev‐Gentofte Hospital Copenhagen Denmark; ^3^ Baker Heart and Diabetes Institute Melbourne Australia; ^4^ Monash University Melbourne Australia; ^5^ Department of Cardiology Rigshospitalet, University of Copenhagen Copenhagen Denmark; ^6^ Department of Cardiology Odense University Hospital Odense Denmark

**Keywords:** Heart failure with preserved ejection fraction, Cardiometabolic, Lactate

## Abstract

**Aims:**

Heart failure with preserved ejection fraction (HFpEF) is associated with an array of central and peripheral haemodynamic and metabolic changes. The exact pathogenesis of exercise limitation in HFpEF remains uncertain. Our aim was to compare lactate accumulation and central haemodynamic responses to exercise in patients with HFpEF, non‐cardiac dyspnoea (NCD), and healthy volunteers.

**Methods and results:**

Right heart catheterization with mixed venous blood gas and lactate measurements was performed at rest and during symptom‐limited supine exercise. Multivariable analyses were conducted to determine the relationship between haemodynamic and biochemical parameters and their association with exercise capacity. Of 362 subjects, 198 (55%) had HFpEF, 103 (28%) had NCD, and 61 (17%) were healthy volunteers. This included 139 (70%) females with HFpEF, 77 (75%) in NCD (*P* = 0.41 HFpEF vs. NCD), and 31 (51%) in healthy volunteers (*P* < 0.001 HFpEF vs. volunteers). The median age was 71 (65, 75) years in HFpEF, 66 (57, 72) years in NCD, and 49 (38, 65) years in healthy volunteers (HFpEF vs. NCD or volunteer, both *P* < 0.001). Peak workload was lower in HFpEF compared with healthy volunteers [52 W (interquartile range 31–73), 150 W (125–175), *P* < 0.001], but not NCD [53 W (33, 75), *P* = 0.85]. Exercise lactate indexed to workload was higher in HFpEF at 0.08 mmol/L/W (0.05–0.11), 0.06 mmol/L/W (0.05–0.08; *P* = 0.016) in NCD, and 0.04 mmol/L/W (0.03–0.05; *P* < 0.001) in volunteers. Exercise cardiac index was 4.5 L/min/m^2^ (3.7–5.5) in HFpEF, 5.2 L/min/m^2^ (4.3–6.2; *P* < 0.001) in NCD, and 9.1 L/min/m^2^ (8.0–9.9; *P* < 0.001) in volunteers. Oxygen delivery in HFpEF was lower at 1553 mL/min (1175–1986) vs. 1758 mL/min (1361–2282; *P* = 0.024) in NCD and 3117 mL/min (2667–3502; *P* < 0.001) in the volunteer group during exercise. Predictors of higher exercise lactate levels in HFpEF following adjustment included female sex and chronic kidney disease (both *P* < 0.001).

**Conclusions:**

HFpEF is associated with reduced exercise capacity secondary to both central and peripheral factors that alter oxygen utilization. This results in hyperlactataemia. In HFpEF, plasma lactate responses to exercise may be a marker of haemodynamic and cardiometabolic derangements and represent an important target for future potential therapies.

## Introduction

Heart failure with preserved ejection fraction (HFpEF) is a heterogeneous condition prevalent in patients with multiple comorbidities. HFpEF is rapidly becoming the predominant heart failure phenotype.^1^ The current landscape of therapeutic failure of heart failure with reduced ejection fraction (HFrEF) medical therapy in HFpEF (with exception of sodium‐glucose cotransporter 2 inhibitors) has been attributed to this physiologic heterogeneity.^2^, ^3^, ^4^, ^5^ This group appears physiologically distinct from patients with low and mid‐range ejection fractions.

Dyspnoea is the most common presenting symptom in HFpEF, but dyspnoea is a highly prevalent and non‐specific symptom and can be due to non‐cardiac causes.^6^ Invasive haemodynamic testing, particularly with exercise, is the cornerstone of understanding the physiological changes associated with HFpEF as well as differentiating from other causes of dyspnoea. Unravelling HFpEF pathophysiology is complex given the interplay of diastolic dysfunction, pulmonary hypertension, microvascular inflammation, and cardiometabolic alterations.^7^ Understanding the HFpEF phenotype and distinguishing it from non‐cardiac dyspnoea (NCD) have important structural, functional, and treatment implications. In particular, the role of lactate both as a measure of cardiometabolic derangement and potentially as a contributor to symptomatology has not been comprehensively studied in HFpEF.

Our aim was to compare the haemodynamic and lactate responses of patients with HFpEF, NCD, and healthy asymptomatic volunteers at rest and exercise to further interrogate differences in physiological phenotypes. We hypothesized that the plasma lactate response to exercise is a marker of cardiometabolic derangement in HFpEF patients.

## Methods

This retrospective observation study included patients who underwent exercise right heart catheterization for investigation of exertional dyspnoea [New York Heart Association (NYHA) class II–IV symptoms] between July 2012 and August 2021. HFpEF was defined as a left ventricular ejection fraction ≥ 50% with an elevated pulmonary capillary wedge pressure (PCWP) at rest of ≥15 mmHg or during exercise ≥25 mmHg.^8^ NCD was defined as dyspnoea of NYHA class II–IV severity not associated with cardiac disease (including valvular disease, cardiomyopathy, ischaemia, and arrhythmia) and not meeting HFpEF criteria. An external cohort of healthy volunteer subjects examined in two centres in Denmark was utilized as a further comparator.^9^ This study was approved by the institutional ethics committees, and all subjects gave informed consent.

### Exercise right heart catheterization

Right heart catheterization was performed at rest and during exercise via supine cycle ergometry. A stepwise exercise protocol was utilized, with an endpoint of symptom‐limited maximal effort, as previously described.^10^ End expiratory rest and exercise measurements of right atrial pressure, pulmonary artery pressure, and PCWP were recorded. Cardiac output was measured via thermodilution. Mixed venous blood gas samples, including lactate, were collected from the pulmonary artery at baseline and peak exercise. Healthy volunteer subjects underwent a similar protocol with measurements taken at 75% predicted maximum oxygen consumption (VO_2_) during upright ergometry, also previously described.^9^

The Fick equation utilized cardiac output, pulse oximetry, and mixed venous results to calculate VO_2_ at rest and during exercise. Standard formulae were used to calculate arteriovenous oxygen content difference, stroke volume (SV), pulmonary and systemic vascular resistance (SVR), oxygen delivery, and oxygen extraction. Values were indexed by body surface area and workload (W), where appropriate.

### Statistical analysis

The data are presented as the median and interquartile range. Analyses between groups were performed using the Fisher exact, *χ* ^2^, and Mann–Whitney *U* tests as appropriate. The predictors of lactate responses in HFpEF were analysed via multivariable regression, including age, sex, body mass index (BMI), atrial fibrillation (AF), diabetes, hypertension, chronic obstructive pulmonary disease (COPD), ischaemic heart disease (IHD), and chronic kidney disease (CKD). To compare responses to exercise, a repeated measures ANOVA with factors time, group, and their interaction was performed, with Kruskal–Wallis post hoc comparisons as appropriate. Two‐sided *P* values < 0.05 were considered statistically significant. Statistical analysis was performed using Stata 17.0 (StataCorp LP, College Station, TX, USA) software and Prism 9.3.1 (GraphPad Software).

## Results

### Baseline characteristics

The study cohort included 198 (55%) with a diagnosis of HFpEF, 103 (28%) with NCD, and 61 (17%) were asymptomatic healthy subjects (*Table* *1*). Patients with HFpEF were older, with a median age of 71 (65, 75) years, compared with NCD and healthy volunteer groups (HFpEF vs. NCD or volunteer, both *P* < 0.001; *Table* *1*). Patients with HFpEF were predominately female [139 (70%)] and had a higher BMI [31 kg/m^2^ (28, 35)] compared with volunteers (both *P* < 0.001; *Table* *1*). HFpEF patients had higher rates of comorbidities, including AF, hypertension, and IHD, compared with NCD.

**Table 1:** Baseline characteristics

	HFpEF	NCD	*P* value	Healthy volunteers	*P* value
	*n* = 198	*n* = 103	HFpEF vs. NCD	*n* = 61	HFpEF vs. volunteers
Age, years	71 (65, 75)	66 (57, 72)	<0.001	49 (38, 65)	<0.001*
Female, *n* (%)	139 (70)	77 (75)	0.41	31 (51)	0.005*
BMI, kg/m^2^	31 (28, 35)	29 (26, 35)	0.075	25 (22, 27)	<0.001*
NYHA, *n* (%)					
II	111 (56)	63 (61)	0.45		
III	85 (43)	40 (39)			
IV	2 (1)	0			
Comorbidities, *n* (%)					
Atrial fibrillation	105 (53)	24 (23)	<0.001		
Hypertension	142 (72)	53 (52)	<0.001		
Diabetes	34 (17)	13 (13)	0.30		
IHD	38 (19)	10 (9.7)	0.033		
COPD	21 (11)	8 (7.7)	0.43		
CKD	25 (16)	6 (8)	0.10	4 (8)	0.16
Smoking, *n* (%)	72 (47)	33 (45)	0.76		
Medications, *n* (%)					
ACE‐I/ARB	113 (57)	38 (37)	0.001		
Beta‐blocker	103 (53)	22 (22)	<0.001		
MRA	33 (17)	10 (9.8)	0.098		
Digoxin	22 (11)	6 (5.8)	0.134		
Diuretic	73 (37)	25 (25)	0.031		
Serum creatinine, μmol/L	75 (65, 94)	72 (63, 85)	0.092		
Haemoglobin, g/L	135 (127, 145)	137 (127, 144)	0.58	140 (134, 151)	<0.001*

ACE‐I, angiotensin‐converting enzyme inhibitor; ARB, angiotensin II receptor blocker; BMI, body mass index; CKD, chronic kidney disease; COPD, chronic obstructive pulmonary disease; HFpEF, heart failure with preserved ejection fraction; IHD, ischaemic heart disease; MRA, mineralocorticoid receptor antagonist; NCD, non‐cardiac dyspnoea; NYHA, New York Heart Association function classification.

Values are represented as the median (interquartile range) or specified.

* Significant *P* < 0.05.

### Baseline haemodynamics

HFpEF was associated with a higher heart rate at baseline compared with healthy volunteers and a higher proportion of AF compared with NCD (*Table* *2*). HFpEF was associated with higher mean arterial pressure (MAP) compared with NCD and volunteers (HFpEF vs. NCD, *P* = 0.039; HFpEF vs. volunteers, *P* < 0.001). HFpEF was associated with a higher SVR compared with volunteers at rest (*P* = 0.003; *Table* *2*). HFpEF was associated with raised right atrial and wedge‐driven pulmonary hypertension at rest compared with NCD and volunteers (*Table* *2*). Cardiac index was lower at rest in HFpEF compared with NCD and the volunteer group (*Table* *2*). Resting VO_2_ was lower in HFpEF compared with volunteers but not NCD. Mixed venous oxygen saturation was lower in HFpEF compared with NCD and volunteers (HFpEF vs. NCD, *P* = 0.006; HFpEF vs. volunteers, *P* < 0.001). Oxygen extraction was higher at 28% in HFpEF compared with NCD and volunteers (HFpEF vs. NCD, *P* < 0.001; HFpEF vs. volunteers, *P* < 0.002).

**Table 2:** Baseline haemodynamics

	HFpEF	NCD	*P* value	Healthy volunteers	*P* value
	*n* = 198	*n* = 103	HFpEF vs. NCD	*n* = 61	HFpEF vs. volunteers
Heart rate, b.p.m.	66 (60, 75)	69 (62, 79)	0.154	63 (58, 68)	0.007*
MAP, mmHg	100 (90, 109)	96 (88, 104)	0.039*	89 (81, 98)	<0.001*
Resting atrial fibrillation	49 (27)	8 (9.2)	0.001*		
Resting right heart catheterization					
RAP, mmHg	7 (5, 9)	4 (3, 6)	<0.001*	5 (4, 7)	<0.001*
Mean PAP, mmHg	21 (18, 26)	17 (14, 20)	<0.001*	14 (12, 16)	<0.001*
PCWP, mmHg	13 (10, 16)	8 (6, 10)	<0.001*	9 (7, 10)	<0.001*
Cardiac index, L/min/m^2^	2.6 (2.2, 3.0)	2.7 (2.3, 3.2)	0.018*	2.7 (2.4, 2.9)	0.16
Indexed stroke volume, mL/m^2^	40 (32, 46)	40 (35, 45)	0.51	44 (39, 49)	0.001*
SVR, mmHg·min/L	18 (15, 21)	19 (15, 21)	0.41	16 (14, 19)	0.003*
PVR, mmHg·min/L	1.8 (1.3, 2.2)	1.5 (1.1, 2.1)	0.23	1.1 (0.8, 1.4)	<0.001*
Mixed venous oxygen saturation, %	71 (69, 75)	73 (70, 77)	0.006*	74 (71, 77)	<0.001*
Arteriovenous oxygen difference, mL/100 mL	5 (4.4, 5.8)	4.4 (3.9, 5.1)	<0.001*	4.5 (4.1, 4.9)	0.068
VO_2_, mL/kg/min	2.7 (2.4, 3.1)	2.8 (2.5, 3.2)	0.75	3.1 (2.6, 3.6)	0.006*
Oxygen delivery, mL/min	880 (726, 1041)	964 (747, 1194)	0.012*	932 (811, 1036)	0.22
Oxygen extraction ratio, %	28 (24, 31)	25 (22, 28)	<0.001*	24 (22, 27)	<0.002*
Lactate, mmol/L	1.1 (0.9, 1.4)	1.1 (0.9, 1.4)	0.82	0.6 (0.4, 0.8)	<0.001*

HFpEF, heart failure with preserved ejection fraction; MAP, mean arterial pressure; NCD, non‐cardiac dyspnoea; PAP, pulmonary artery pressure; PCWP, pulmonary capillary wedge pressure; PVR, pulmonary vascular resistance; RAP, right atrial pressure; SVR, systemic vascular resistance; VO_2_, oxygen consumption.

Values are represented as the median (interquartile range) or specified.

* Significant *P* < 0.05.

### Exercise haemodynamics

**Figure 1:**
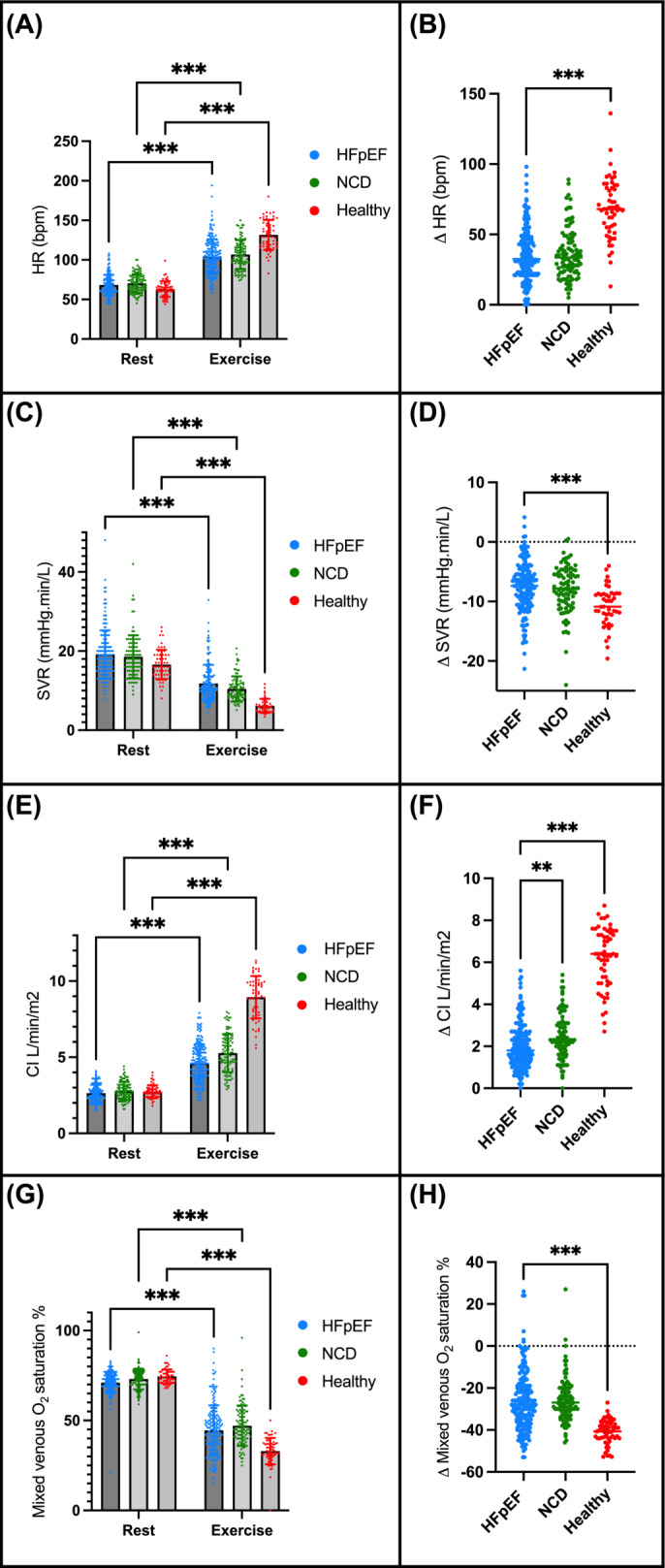
Drivers of lactate responses. (A) Heart rate (HR) at rest, exercise, and (B) change in response to exercise; (C) systemic vascular resistance (SVR) at rest, exercise, and (D) change in response to exercise; (E) cardiac index (CI) at rest, exercise, and (F) change in response to exercise; and (G) mixed venous oxygen saturation at rest, exercise, and (H) change in response to exercise. HFpEF, heart failure with preserved ejection fraction; NCD, non‐cardiac dyspnoea. **P < 0.01, ***P < 0.001.

Peak workload was lower in HFpEF compared with the highest exercise level in healthy volunteers [52 W (31, 73), 150 W (125, 175), *P* < 0.001; *Table* *3*], but not NCD. Heart rate increased with exercise but did not augment at peak workload to the same extent in HFpEF compared with volunteers (*P* < 0.001; *Figure* *1* *A/B*). HFpEF patients had higher exercise MAP and SVR compared with NCD patients and volunteers (*Table* *3*). SVR decreased with exercise to a lesser extent in HFpEF as compared with volunteers (*Figure* *1* *C/D*). Exercise in HFpEF was associated with raised right atrial and wedge‐driven pulmonary pressures compared with NCD and volunteers. Exercise cardiac index was 4.5 L/min/m^2^ (3.7, 5.5) in HFpEF, 5.2 L/min/m^2^ (4.3, 6.2; *P* < 0.001) in NCD, and 9.1 L/min/m^2^ (8.0, 9.9; *P* < 0.001) in volunteers. There was a significant increase in cardiac index with exercise, with a greater increase in volunteers and NCD compared with HFpEF (*Figure* *1* *E/F*). Exercise VO_2_ was significantly lower in HFpEF [9.8 mL/kg/min (7.5, 12.5)] compared with NCD [10.5 mL/kg/min (9, 14.4), *P* = 0.038] and volunteers [28.5 mL/kg/min (23.3, 31.2), *P* < 0.001]. VO_2_‐indexed workload was lower in HFpEF compared with NCD but not in volunteers. Mixed venous oxygen saturation was higher in HFpEF compared with volunteers and did not decrease to the same degree during exercise (*P* < 0.001; *Figure* *1* *G/H*). During exercise, oxygen delivery was lower in HFpEF compared with NCD and volunteers, and oxygen extraction was also lower in HFpEF compared with volunteers (*Table* *3*).

**Table 3:** Haemodynamic parameters with exercise

	HFpEF	NCD	*P* value	Healthy volunteers	*P* value
	*n* = 198	*n* = 103	HFpEF vs. NCD	*n* = 61	HFpEF vs. volunteers
Peak work, W	52 (31, 73)	53 (33, 75)	0.85	150 (125, 175)	<0.001*
Heart rate, b.p.m.	103 (88, 118)	106 (96, 119)	0.30	129 (118. 147)	<0.001*
MAP, mmHg	114 (103, 125)	105 (96, 115)	0.001	108 (95, 118)	0.005
Right heart catheterization					
RAP, mmHg	16 (13, 19)	9 (6, 12)	<0.001*	8 (6, 12)	<0.001*
Mean PAP, mmHg	43 (38, 48)	33 (27, 37)	<0.001*	31 (25, 40)	<0.001*
PCWP, mmHg	30 (26, 33)	18 (14, 21)	<0.001*	17 (11, 24)	<0.001*
Cardiac index, L/min/m^2^	4.5 (3.7, 5.5)	5.2 (4.3, 6.2)	<0.001*	9.1 (8.0, 9.9)	<0.001*
Indexed stroke volume, mL/m^2^	46 (37, 52)	49 (42, 55)	0.001*	68 (62, 76)	<0.001*
SVR, mmHg·min/L	10.6 (8.7, 13.7)	9.7 (8.2, 11.9)	0.014*	5.9 (4.8, 7.0)	<0.001*
PVR, mmHg·min/L	1.4 (1.0, 2.0)	1.4 (0.9, 2.0)	0.77	0.7 (0.6, 1.0)	<0.001*
Mixed venous oxygen saturation, %	44 (34, 52)	45 (40, 54)	0.10	33 (30, 37)	<0.001*
Arteriovenous oxygen difference, mL/100 mL	9.8 (8.5, 11.6)	9.3 (8.1, 10.5)	0.043*	12.1 (11.3, 12.9)	<0.001*
VO_2_, mL/kg/min	9.8 (7.5, 12.5)	10.5 (9, 14.4)	0.038*	28.5 (23.3, 31.2)	<0.001*
VO_2_‐indexed workload, mL/kg/min/W	0.19 (0.14, 0.25)	0.21 (0.17, 0.29)	0.023*	0.20 (0.17, 0.23)	0.57
Oxygen delivery, mL/min	1553 (1175, 1986)	1758 (1361, 2282)	0.024*	3117 (2667, 3502)	<0.001*
Oxygen extraction ratio, %	55 (46, 64)	53 (44, 57)	0.062	66 (62, 69)	<0.001*
Lactate, mmol/L	3.7 (2.7, 5.2)	3.3 (2.3, 4.4)	0.009*	5.2 (4.2, 7.4)	<0.001*
Lactate‐indexed workload, mmol/L/W	0.08 (0.05, 0.11)	0.06 (0.05, 0.08)	0.016*	0.04 (0.03, 0.05)	<0.001*

HFpEF, heart failure with preserved ejection fraction; MAP, mean arterial pressure; NCD, non‐cardiac dyspnoea; PAP, pulmonary artery pressure; PCWP, pulmonary capillary wedge pressure; PVR, pulmonary vascular resistance; RAP, right atrial pressure; SVR, systemic vascular resistance; VO_2_, oxygen consumption.

Values are represented as the median (interquartile range) or specified.

* Significant *P* < 0.05.

### Lactate responses

**Figure 2:**
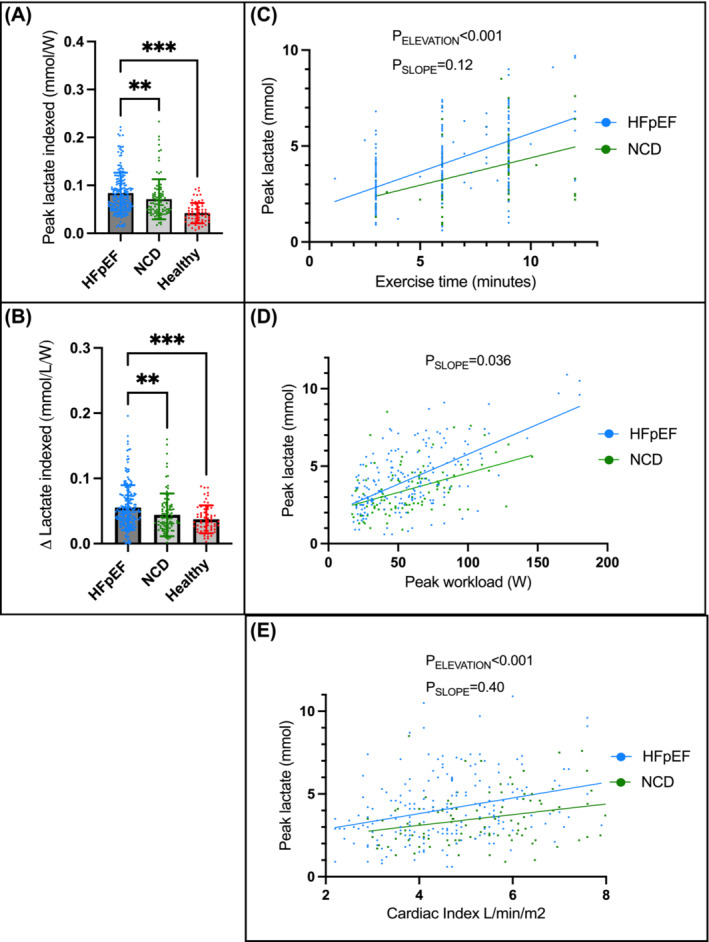
Lactate responses to exercise. (A) Peak lactate indexed to workload; (B) change in lactate indexed to workload; (C) peak lactate with exercise time; (D) peak lactate with peak workload; and (E) peak lactate with peak vs. peak cardiac index. There was no association between peak workload and peak lactate in healthy volunteers, and time data were not available for volunteers. It was not possible to determine elevation in (D) because the slopes differ so much. HFpEF, heart failure with preserved ejection fraction; NCD, non‐cardiac dyspnoea. **P < 0.01, ***P < 0.001.

Resting lactate in HFpEF was 1.1 mmol/L (0.9, 1.4) compared with 0.6 mmol/L (0.4, 0.8) in healthy volunteers (*P* < 0.001; *Table* *3*). There was no difference in comparison with NCD (*P* = 0.82). Exercise lactate indexed to workload in HFpEF was 0.08 mmol/L/W (0.05, 0.11), higher than 0.06 mmol/L/W (0.05, 0.08; *P* = 0.016) in NCD, and 0.04 mmol/L/W (0.03, 0.05; *P* < 0.001) in healthy volunteers (*Figure* *2* *A*). There was a greater increase in lactate in HFpEF compared with NCD and volunteers (*Figure* *2* *B*). At any given duration of exercise, peak workload, or cardiac index, patients with HFpEF had higher lactate compared with NCD (*Figure* *2* *C/D/E*).

In HFpEF, multivariable analysis demonstrated that higher BMI > 30 and diabetes were associated with higher resting lactate levels {BMI: 0.16 mmol [95% confidence interval (CI) 0.03, 0.29], *P* = 0.014; diabetes: 0.32 mmol [95% CI 0.14, 0.49], *P* < 0.001; *Table* *4*}. At peak exercise in HFpEF, predictors of indexed lactate levels included female sex and CKD, with age approaching significance [female: 0.03 mmol/W (95% CI 0.01, 0.04), *P* < 0.001; CKD: 0.03 mmol/W (95% CI 0.01, 0.05), *P* < 0.001; *Table* *5*].

**Table 4:** Multivariable predictors of resting lactate in heart failure with preserved ejection fraction

	Coefficient	95% CI	*P* value
Age	0.002	−0.005, 0.009	0.56
BMI > 30	0.16	0.03, 0.29	0.014*
Female	−0.07	−0.22, 0.08	0.35
Atrial fibrillation	−0.001	−0.13, 0.14	0.99
Diabetes	0.32	0.14, 0.49	<0.001*
Hypertension	0.02	−0.13, 0.17	0.83
COPD	0.06	−0.15, 0.27	0.57
CKD	0.05	−0.14, 0.25	0.56
IHD	−0.04	−0.22, 0.13	0.63

BMI, body mass index; CI, confidence interval; CKD, chronic kidney disease; COPD, chronic obstructive pulmonary disease; IHD, ischaemic heart disease.

* Significant *P* < 0.05.

**Table 5:** Multivariable predictors of exercise lactate indexed to workload in heart failure with preserved ejection fraction

	Coefficient	95% CI	*P* value
Age	0.001	0.00, 0.001	0.059
BMI > 30	−0.003	−0.01, 0.01	0.64
Female	0.03	0.01, 0.04	<0.001*
Atrial fibrillation	0.002	−0.01, 0.01	0.71
Diabetes	0.01	−0.01, 0.02	0.38
Hypertension	0.01	−0.045, 0.02	0.18
COPD	0.01	−0.01, 0.02	0.49
CKD	0.03	0.01, 0.05	<0.001*
IHD	0.01	−0.01, 0.02	0.33

BMI, body mass index; CI, confidence interval; CKD, chronic kidney disease; COPD, chronic obstructive pulmonary disease; IHD, ischaemic heart disease.

* Significant *P* < 0.05.

## Discussion

The key finding of this study was that HFpEF was associated with a reduced augmentation of oxygen delivery in response to exercise, as reflected by the degree of lactataemia. We demonstrated a profile of volume expansion as evidenced by raised right atrial pressures, haemodynamic evidence of wedge‐driven pulmonary hypertension, and reduced cardiac output, as well as altered oxygen dynamics associated with cardiometabolic changes—raising lactate levels. The culmination of these effects led to the marked exercise intolerance observed in patients with HFpEF.^7^ Thus, the plasma lactate response to exercise may be a marker of cardiometabolic derangement in HFpEF patients. Further randomized studies are required to substantiate these findings.

We demonstrated higher resting lactate in HFpEF patients and a significant increase in indexed lactate from baseline during exercise in HFpEF compared with healthy volunteers. Key central physiological changes in HFpEF likely contribute to lactataemia. We and others have demonstrated that HFpEF is associated with chronotropic incompetence.^11^, ^12^ In the current study, cardiac index was lower in HFpEF during exercise, a product of reduced SV likely secondary to diastolic dysfunction and a lower heart rate. HFpEF was further associated with changes in peripheral oxygen dynamics with a higher mixed venous oxygen saturation, reduced oxygen delivery, and reduced oxygen extraction ratio. Raised lactate levels in this setting may demonstrate an earlier transition to anaerobic metabolism, given lower cardiac output and oxygen delivery to tissue. Previous studies have demonstrated altered energetics not only in the heart but also in the periphery. For example, oxygen utilization in the skeletal muscle of older HFpEF patients was shown to correlate with reduced exercise capacity.^13^ Several studies have proposed that non‐cardiac factors in HFpEF exercise capacity may contribute to peripheral lactate accumulation. These factors include altered mitochondrial content and oxidative capacity, reduced type 1 oxidative fibres, and greater anaerobic reliance, which have potential as therapeutic targets.^13^, ^14^, ^15^, ^16^

Shifting paradigms now place less evidence on an anaerobic or lactate threshold, rather emphasizing the role of lactate as a substrate, precursor in gluconeogenesis, and signalling molecule.^17^, ^18^ Thus, early lactate accumulation during exercise may signal diminished capacity in HFpEF patients to respond to this stressor, with impaired ability to utilize lactate as an energy substrate. An imbalance between lactate production and removal may be explained by the altered skeletal muscle composition and metabolism in HFpEF, leading to reduced lactate oxidation in working muscle, and congestive hepatopathy may further reduce lactate clearance.^14^ Lactate is an established signalling molecule and a key substrate for cardiac myocytes.^19^ HFpEF has also been associated with changes to cardiac myocyte mitochondrial structure and function, altering lactate homeostasis and calcium handling.^20^ The role of lactate in HFpEF is evolving, but given that lactataemia occurred at a relatively low peak work level and with a small increase in cardiac index, this represents a significant finding. These patients may have frequent and prolonged lactataemia from daily activities alone, and further investigation into the potential consequences of this is warranted. It also indicates that targeting bioenergetic pathways, such as sodium‐glucose cotransporter 2 inhibitors, to correct the heart's capacity to generate energy should be explored in HFpEF.^21^

Patients with HFpEF and NCD did not differ in severity of symptoms, and both achieved similarly low peak workloads. NCD is an important comparator in HFpEF; both groups present with significant dyspnoea impacting their daily activities. Differentiating symptomatology from the underlying pathological process is crucial to better understanding HFpEF and guiding specific treatment. HFpEF was associated with a greater degree of volume overload and wedge‐driven pulmonary hypertension at rest and during exercise, again demonstrating the diagnostic differences compared with NCD. Additionally, HFpEF was associated with altered peripheral oxygen handling at rest, reflecting a higher basal metabolic rate. HFpEF was associated with a higher serum lactate for any given exercise time, workload, or cardiac index compared with NCD. This suggests that beyond the role of central factors, key peripheral factors, including altered peripheral vascular function, inflammation, and altered mitochondrial metabolism, are involved.^16^ There is an ongoing need for effective therapies targeting both central haemodynamic changes (chronotropic incompetence and contractility) and the complex and interconnected peripheral changes that contribute to the HFpEF phenotype.

Adding to the complexity of HFpEF is the identification of different subpopulations—AF, age, sex, and diabetes have been demonstrated to further alter HFpEF haemodynamics.^22^, ^23^ In our study, analyses of factors predicting change in lactate indicated that resting lactate was impacted by diabetes and obesity, two highly prevalent comorbidities. Diabetes is associated with raised basal lactate levels secondary to altered oxidative capacity and structural changes in skeletal muscle.^24^, ^25^ Obesity is also associated with reduced functional reserve, a more severe HFpEF phenotype, and an array of metabolic and inflammatory derangements that likely contribute to lactataemia.^26^ In the current study, at peak exercise, female sex and CKD were associated with higher lactate levels. Females have been previously shown to have higher peak lactate levels, which may be due to a lower ventilatory threshold compared with males.^23^ CKD is an independent predictor of poor outcomes in HFpEF and a key site of lactate removal.^27^, ^28^ HFpEF is multifactorial with crossover with multiple comorbidities, which highlights the importance of optimizing concurrent medical conditions when making treatment decisions.

This study was retrospective, but it did have the advantage of invasive haemodynamic testing to confirm the diagnosis of HFpEF. In this study, arterial blood gases were not available, and thus, oxygen consumption calculations were derived based on pulse oximetry. Right heart catheterization in healthy subjects is uncommon. Here, we utilized data from healthy asymptomatic subjects as a comparator to exemplify the impact of HFpEF. However, it should be noted that differences in the baseline characteristics between these populations were observed; for example, volunteers were younger, more often male, and had a lower BMI. Volunteer subjects were examined during exercise via upright ergometry (not supine) at 75% of maximal predicted VO_2_ and not maximal VO_2_, but significant differences were observed compared with HFpEF and NCD despite this.

We have demonstrated that HFpEF is associated with lactataemia, which may be a marker of the haemodynamic and cardiometabolic derangements in this syndrome. HFpEF is associated with reduced exercise capacity secondary to both central and peripheral factors that alter oxygen utilization. This alteration results in higher lactate levels. This contributes to the growing body of evidence that HFpEF pathophysiology goes beyond diastolic dysfunction and represents a multisystem inflammatory and metabolic phenotype. The focus should shift to the development of therapies that target peripheral and metabolic factors in HFpEF, given the minimal efficacy to date of centrally acting agents.

## Conflict of interest

F.G.: advisor: Bayer, Abbott, FineHeart, Pfizer, Alnylam, AstraZeneca, and Ionis outside the current work; speaker: Orion Pharma, Vifor, and Novartis outside the current work. J.E.M.: steering committee and research grant: Abiomed; speaker: Boehringer Ingelheim, Orion, Abiomed, and Abbott, all outside the submitted work. E.W.: speaker: AstraZeneca, Boehringer Ingelheim, and Novartis; advisor: Boehringer Ingelheim, Novartis, and Bayer.

## Funding

Nil.

## References

[1] Dunlay SM , Roger VL , Redfield MM . Epidemiology of heart failure with preserved ejection fraction. Nat Rev Cardiol 2017;14:591‐602. doi:10.1038/nrcardio.2017.65 28492288

[2] Yusuf S , Pfeffer MA , Swedberg K , Granger CB , Held P , McMurray JJ , *et al*. Effects of candesartan in patients with chronic heart failure and preserved left‐ventricular ejection fraction: The CHARM‐Preserved trial. Lancet 2003;362:777‐781. doi:10.1016/S0140-6736(03)14285-7 13678871

[3] Solomon SD , McMurray JJV , Committee P‐HS, Investigators . Angiotensin‐neprilysin inhibition in heart failure with preserved ejection fraction. Reply. N Engl J Med 2020;382:1182‐1183. doi:10.1056/NEJMc2000284 32187481

[4] Solomon SD , McMurray JJV , Claggett B , de Boer RA , DeMets D , Hernandez AF , *et al*. Dapagliflozin in heart failure with mildly reduced or preserved ejection fraction. N Engl J Med 2022;387:1089‐1098. doi:10.1016/j.jchf.2021.11.006 36027570

[5] Anker SD , Butler J , Filippatos G , Ferreira JP , Bocchi E , Bohm M , *et al*. Empagliflozin in heart failure with a preserved ejection fraction. N Engl J Med 2021;385:1451‐1461. doi:10.1056/NEJMoa2107038 34449189

[6] Bhatia RS , Tu JV , Lee DS , Austin PC , Fang J , Haouzi A , *et al*. Outcome of heart failure with preserved ejection fraction in a population‐based study. N Engl J Med 2006;355:260‐269. doi:10.1056/NEJMoa051530 16855266

[7] Lam CSP , Voors AA , de Boer RA , Solomon SD , van Veldhuisen DJ . Heart failure with preserved ejection fraction: From mechanisms to therapies. Eur Heart J 2018;39:2780‐2792. doi:10.1093/eurheartj/ehy301 29905796

[8] Borlaug BA , Nishimura RA , Sorajja P , Lam CS , Redfield MM . Exercise hemodynamics enhance diagnosis of early heart failure with preserved ejection fraction. Circ Heart Fail 2010;3:588‐595. doi:10.1161/CIRCHEARTFAILURE.109.930701 20543134 PMC3048586

[9] Wolsk E , Bakkestrom R , Thomsen JH , Balling L , Andersen MJ , Dahl JS , *et al*. The influence of age on hemodynamic parameters during rest and exercise in healthy individuals. JACC Heart Fail 2017;5:337‐346. doi:10.1016/j.jchf.2016.10.012 28017352

[10] Maeder MT , Thompson BR , Brunner‐La Rocca HP , Kaye DM . Hemodynamic basis of exercise limitation in patients with heart failure and normal ejection fraction. J Am Coll Cardiol 2010;56:855‐863. doi:10.1016/j.jacc.2010.04.040 20813283

[11] Borlaug BA , Melenovsky V , Russell SD , Kessler K , Pacak K , Becker LC , *et al*. Impaired chronotropic and vasodilator reserves limit exercise capacity in patients with heart failure and a preserved ejection fraction. Circulation 2006;114:2138‐2147. doi:10.1161/CIRCULATIONAHA.106.632745 17088459

[12] Wolsk E , Kaye DM , Komtebedde J , Shah SJ , Borlaug BA , Burkhoff D , *et al*. Determinants and consequences of heart rate and stroke volume response to exercise in patients with heart failure and preserved ejection fraction. Eur J Heart Fail 2021;23:754‐764. doi:10.1002/ejhf.2146 33686716

[13] Haykowsky MJ , Kouba EJ , Brubaker PH , Nicklas BJ , Eggebeen J , Kitzman DW . Skeletal muscle composition and its relation to exercise intolerance in older patients with heart failure and preserved ejection fraction. Am J Cardiol 2014;113:1211‐1216. doi:10.1016/j.amjcard.2013.12.031 24507172 PMC4282135

[14] Molina AJ , Bharadwaj MS , Van Horn C , Nicklas BJ , Lyles MF , Eggebeen J , *et al*. Skeletal muscle mitochondrial content, oxidative capacity, and Mfn2 expression are reduced in older patients with heart failure and preserved ejection fraction and are related to exercise intolerance. JACC Heart Fail 2016;4:636‐645. doi:10.1016/j.jchf.2016.03.011 27179829 PMC4967040

[15] Bhella PS , Prasad A , Heinicke K , Hastings JL , Arbab‐Zadeh A , Adams‐Huet B , *et al*. Abnormal haemodynamic response to exercise in heart failure with preserved ejection fraction. Eur J Heart Fail 2011;13:1296‐1304. doi:10.1093/eurjhf/hfr133 21979991 PMC3220394

[16] Deng Y , Xie M , Li Q , Xu X , Ou W , Zhang Y , *et al*. Targeting mitochondria‐inflammation circuit by β‐hydroxybutyrate mitigates HFpEF. Circ Res 2021;128:232‐245. doi:10.1161/CIRCRESAHA.120.317933 33176578

[17] Poole DC , Rossiter HB , Brooks GA , Gladden LB . The anaerobic threshold: 50+ years of controversy. J Physiol 2021;599:737‐767. doi:10.1113/JP279963 33112439

[18] Brooks GA . Cell‐cell and intracellular lactate shuttles. J Physiol 2009;587:5591‐5600. doi:10.1113/jphysiol.2009.178350 19805739 PMC2805372

[19] Stanley WC . Myocardial lactate metabolism during exercise. Med Sci Sports Exerc 1991;23:920‐924. 1956265

[20] Lopaschuk GD . Metabolic modulators in heart disease: Past, present, and future. Can J Cardiol 2017;33:838‐849. doi:10.1016/j.cjca.2016.12.013 28279520

[21] Maejima Y . SGLT2 inhibitors play a salutary role in heart failure via modulation of the mitochondrial function. Front Cardiovasc Med 2019;6:186. doi:10.3389/fcvm.2019.00186 31970162 PMC6960132

[22] Nanayakkara S , Haykowsky M , Mariani J , Van Empel V , Maeder MT , Vizi D , *et al*. Hemodynamic profile of patients with heart failure and preserved ejection fraction vary by age. J Am Heart Assoc 2017;6: doi:10.1161/JAHA.116.005434 PMC5634249 28939710

[23] Beale AL , Nanayakkara S , Segan L , Mariani JA , Maeder MT , van Empel V , *et al*. Sex differences in heart failure with preserved ejection fraction pathophysiology: A detailed invasive hemodynamic and echocardiographic analysis. JACC Heart Fail 2019;7:239‐249. doi:10.1016/j.jchf.2019.01.004 30819380

[24] Lovejoy J , Newby FD , Gebhart SS , DiGirolamo M . Insulin resistance in obesity is associated with elevated basal lactate levels and diminished lactate appearance following intravenous glucose and insulin. Metabolism 1992;41:22‐27. doi:10.1016/0026-0495(92)90185-d 1538640

[25] Lindman BR , Davila‐Roman VG , Mann DL , McNulty S , Semigran MJ , Lewis GD , *et al*. Cardiovascular phenotype in HFpEF patients with or without diabetes: A RELAX trial ancillary study. J Am Coll Cardiol 2014;64:541‐549. doi:10.1016/j.jacc.2014.05.030 25104521 PMC4133145

[26] Obokata M , Borlaug BA . Response by Obokata and Borlaug to letters regarding article, “Evidence supporting the existence of a distinct obese phenotype of heart failure with preserved ejection fraction”. Circulation 2018;137:416‐417. doi:10.1161/CIRCULATIONAHA.117.031394 29358350 PMC5784768

[27] Kirkman DL , Carbone S , Canada JM , Trankle C , Kadariya D , Buckley L , *et al*. The chronic kidney disease phenotype of HFpEF: Unique cardiac characteristics. Am J Cardiol 2021;142:143‐145. doi:10.1016/j.amjcard.2020.12.012 33333073 PMC8425110

[28] Bellomo R . Bench‐to‐bedside review: Lactate and the kidney. Crit Care 2002;6:322‐326. doi:10.1186/cc1518 12225607 PMC137458

